# Role of serum C1q/TNF-related protein family levels in patients with acute coronary syndrome

**DOI:** 10.3389/fcvm.2022.967918

**Published:** 2022-08-19

**Authors:** Yixiang Liu, Chen Wei, Zhenjiang Ding, Enhong Xing, Zhuoyan Zhao, Fei Shi, Yanan Tian, Ying Zhang, Wenjun Fan, Lixian Sun

**Affiliations:** ^1^Department of Cardiology, Chengde Medical University Affiliated Hospital, Chengde, China; ^2^Central Laboratory, Chengde Medical University Affiliated Hospital, Chengde, China

**Keywords:** C1q/TNF-related protein, acute coronary syndrome, coronary angiography, Gensini score, diagnosis

## Abstract

**Background:**

The C1q/TNF-related protein (CTRP) family affects inflammation regulation, energy metabolism, and insulin signaling. However, their role in acute coronary syndrome (ACS) development is unclear. In this cross-sectional study, we aimed to investigate the association between CTRP family and ACS.

**Methods:**

We enrolled 289 consecutive inpatients with suspected ACS. Serum CTRP family, tumor necrosis factor-α (TNF-α), and adiponectin (ADP) levels were assessed using enzyme-linked immunosorbent assay (ELISA). Multivariate logistic regression and subgroup analyses were used to assess risk factors for ACS. Spearman's tests were used to analyze correlations between CTRP family and continuous variables.

**Results:**

Serum CTRP family levels differed significantly between ACS and Control groups (*p* < 0.05). After adjusting for confounding factors, CTRP family were independently associated with ACS (*p* < 0.05). The association between serum CTRP family levels and ACS was stable in various subgroups according to sex, age, diabetes mellitus, and dyslipidemia status (*p* for interaction > 0.05). Increasing tertiles of serum CTRP1 levels, significantly increased ACS risks, which decreased gradually with increasing CTRP2, CTRP12, and CTRP13 tertiles (*p* for trend < 0.05). Additionally, serum CTRP1, CTRP2, CTRP13, and CTRP15 levels were weakly correlated with the severity of coronary artery stenosis.

**Conclusion:**

CTRP1 and CTRP5 were identified as independent ACS risk factors, whereas CTRP2, CTRP3, CTRP9, CTRP12, CTRP13, and CTRP15 were independent protective factors for ACS. CTRP family, especially CTRP1 and CTRP3 could be novel potential clinical biomarkers of ACS.

## Introduction

Coronary artery disease (CAD), particularly acute coronary syndrome (ACS), is currently the leading cause of cardiovascular mortality and morbidity worldwide, despite numerous advances in diagnosis and treatment (1). As a chronic inflammatory disorder, atherosclerosis is speculated to be the common pathological basis of ACS. The formation, rupture, and erosion of atherosclerotic plaques can cause lumen stenosis and thrombosis, leading to acute cardiovascular events (2). The diagnosis of ACS is mainly based on symptoms, electrocardiogram (ECG), cardiac biomarkers, and coronary angiography (CAG) (3). Therefore, the finding of novel biomarkers for earlier diagnosis and risk assessment of ACS has important clinical implications. Epicardial adipose tissue (EAT) is a fat depot located between the myocardium and visceral layer of the epicardium, which affects the development of ACS *via* paracrine or vasocrine secretion of adipocytokines (4).

C1q/TNF-related protein (CTRP) are a family of adiponectin (ADP) paralogs, including CTRP1 to CTRP15, which show the highest expression in adipose tissue around the heart (5). Recently, increasing evidence suggests that CTRP family have a role in inflammation regulation, energy metabolism, insulin signaling, and cardiovascular system (6). However, the role of CTRP family members as predictive biomarkers for ACS remains unclear, especially, as their causative risk in different ACS subgroups is still uncertain. In the present study, we aimed to investigate the relationship between CTRP family and the prevalence of ACS. In addition, we performed a subgroup analysis to verify the reliability of our conclusions.

## Materials and methods

### Study population

A total of 289 inpatients with suspected ACS were consecutively enrolled for this cross-sectional study from October 2020 to December 2021 at Chengde Medical University Affiliated Hospital. They were divided into the ACS (*n* = 210) and Control (n = 79) groups according to the diagnostic criteria of ACS (7, 8). ACS group: The clinical types of ACS include unstable angina (UA), non-ST-segment elevation myocardial infarction (NSTEMI), and acute ST-segment elevation myocardial infarction (STEMI). UA is defined as myocardial ischemia at rest or on minimal exertion in the absence of acute cardiomyocyte injury or necrosis. Acute myocardial infarction (AMI) is defined as the detection of an increase and/or decrease of a cardiac biomarker, with at least one value above the 99th percentile of the upper reference limit, and with at least one of the following: symptoms of myocardial ischemia, new ischemic ECG changes, development of pathological Q waves on ECG, imaging evidence of loss of viable myocardium, or new regional wall motion abnormality, intracoronary thrombus detected on angiography or autopsy. On this basis, patients with persistent (>20 min) ST-segment elevation in at least two contiguous is defined as STEMI. In contrast, patients without persistent ST-segment elevation are defined as NSTEM. Angiographic ACS is defined as stenosis ≥50% of one or more of the left main, left anterior descending artery, left circumflex artery, right coronary artery, or their main branches. Control group: patients in the Control group were excluded from CAD based on symptoms, ECG, cardiac biomarkers, and CAG. Meanwhile, all of them had <50% luminal stenosis. The study exclusion criteria were: stable coronary artery disease, coronary artery spasm or other secondary angina or myocardial infarction; cardiogenic shock; cardiac arrest; infectious diseases; malignant tumors; and severe heart diseases (e.g., aortic dissection and hypertrophic cardiomyopathy), systemic inflammatory disorders, and hepatic and renal dysfunction. This study was approved by the Institutional Review Board of Chengde Medical University Affiliated Hospital (Number: CYFYLL2022145), and all participants provided written informed consent before participating.

### Baseline demographics and clinical characteristics

Demographics and clinical characteristics, including sex, age, height, weight, body mass index (BMI), history of smoking, dyslipidemia, hypertension, diabetes mellitus (DM), metabolic syndrome (MetS), history of stroke, and family history of CAD, were collected by our research team. Blood pressure at admission were recorded by one single measuring before the CAG. Routine blood test results, and serum biochemistry results, such as total cholesterol (TC), triglycerides (TG), low-density lipoprotein cholesterol (LDL-C), high-density lipoprotein cholesterol (HDL-C), albumin (ALB), creatinine (Cr), homocysteine (Hcy) were also recorded. MetS was defined as the presence of three or more of the following criteria: BMI > 30 kg/m^2^, HDL-C <50 mg/dL in women and <40 mg/dL in men, fasting plasma TG ≥ 150 mg/dL, systolic blood pressure ≥ 130 mmHg, diastolic blood pressure ≥ 85 mmHg, fasting plasma glucose (FPG) ≥ 100 mg/dL or previously diagnosed type 2 DM (T2DM) (9).

### Coronary angiography and Gensini score

The angiogram results were independently evaluated and explained by two experienced interventional cardiologists who were blinded to this study. The severity of coronary artery stenosis was quantitated using the international Gensini score and a guide for score calculation (10).

### Measurement of CTRP family

Blood samples of all patients were drawn from the radial artery before the CAG was performed. The blood samples were centrifuged at 3,000 g for 10 min, and then stored at −80 °C. The serum levels of CTRP1 (MM-2097H1), CTRP2 (MM-60202H1), CTRP3 (MM-1628H1), CTRP5 (MM-610054H1), CTRP9 (MM-2503H1), CTRP12 (MM-50530H1), CTRP13 (MM-60207H1), CTRP15 (MM-60212H1), TNF-α (MM-0122H1), and ADP (MM-1535H1) were measured using an enzyme-linked immunosorbent assay (ELISA) kit (Jiangsu Meimian Industrial Co., Ltd., China).

### Statistical analyses

Statistics analyses were performed using SPSS 26.0 (SPSS Inc, Chicago, IL, USA) and GraphPad Prism 8.0 (GraphPad Software Inc., La Jolla., CA). The sample size was calculated *via* PASS 15 software (NCSS Statistical Software). The Kolmogorov-Smirnov test was used to analyze the continuous variables to verify the normal distribution of variables, and the results were reported as medians and interquartile ranges for skewed distributions. The Mann-Whitney U test was used to compare the relationship of all continuous variables between the ACS and Control groups. Categorical variables were expressed as frequency and percentages, and compared using the chi-squared test.

Receiver-operating characteristic (ROC) curves were used to calculate the cutoff value of adipocytokines for diagnosing ACS. Youden's index (sensitivity + specificity – 1) was calculated, the maximum value which corresponds to the optimal cutoff value of CTRP family members. Multivariable logistic regression models were constructed to evaluate the association between CTRP family and ACS in the general population and different subgroups. The odds ratio (OR) was determined based on 1 standard deviation (1-SD) increases of serum CTRP family levels. In addition, for subgroup analysis, the baseline variables were selected and entered into the multivariable logistic regression models at *p* < 0.1.

To examine the independent performance of CTRP family for assessing the occurrence of ACS, participants were divided into three groups (T1, T2, and T3) according to the tertiles of serum CTRP family levels. We examined the linear trend using *p* for trend, which was calculated using CTRP family tertiles as a continuous variable. Spearman's tests were used to analyze the correlations between CTRP family and continuous variables. A Kruskal-Wallis H test was used to test for differences between the quartiles groups of the Gensini score. The missing data were handled automatically by the statistic software. Two-tailed *p* < 0.05 were considered significant.

## Results

### Baseline clinical characteristics

A total of 289 patients were included in this study and their baseline clinical characteristics are presented in Table 1. The proportion of male patients, those who smoke, and those with dyslipidemia, hypertension, DM, and MetS was higher in the ACS group than in the Control group (*p* < 0.05).

**Table 1:** Baseline clinical characteristics of ACS and control groups.

**Variables**	**ACS group (*n* = 210)**	**Control group (*n* = 79)**	**χ^2^/*Z***	***p*-value**
**Demographics and clinical data**				
Male	142 (67.6)	36 (45.6)	11.798	0.001
Age ≥ 65 years	62 (29.5)	24 (30.4)	0.020	0.887
BMI (kg/m^2^)	25.09 (22.85, 27.94)	24.89 (22.78, 27.53)	−0.439	0.660
Smoking	120 (57.1)	32 (40.5)	6.372	0.012
Dyslipidemia	142 (67.6)	34 (43.0)	14.566	<0.001
Hypertension	127 (60.5)	33 (41.8)	8.127	0.004
DM	83 (39.5)	8 (10.1)	22.996	<0.001
MetS	79 (37.6)	11 (13.9)	15.030	<0.001
History of stroke	41 (19.5)	10 (12.7)	1.862	0.172
Family history of CAD	34 (16.2)	10 (12.7)	0.555	0.456
UA	83 (39.5)			
NSTEMI	55 (26.2)			
STEMI	72 (34.3)			
**Laboratory data**				
WBC count (10^9^/L)	8.55 (6.99, 11.60)	6.81 (5.58, 8.85)	−4.616	<0.001
Platelet count (10^9^/L)	236.00 (196.50, 295.00)	217.00 (174.50, 268.50)	−2.037	0.042
TC (mmol/L)	3.77 (3.03, 4.60)	3.84 (3.24, 4.33)	−0.068	0.946
TG (mmol/L)	1.39 (0.97, 2.00)	1.16 (0.78, 1.62)	−2.572	0.010
LDL-C (mmol/L)	2.11 (1.54, 2.85)	2.24 (1.68, 2.65)	−0.120	0.905
HDL-C (mmol/L)	1.03 (0.84, 1.24)	1.16 (1.03, 1.42)	−4.245	<0.001
ALB (g/L)	40.50 (38.04, 43.10)	42.05 (39.57, 44.89)	−3.049	0.002
Cr (μmol/L)	69.31 (59.25, 80.03)	62.55 (53.84, 70.63)	−3.359	0.001
Serum uric acid (μmol/L)	336.19 (275.39, 402.07)	298.52 (263.54, 360.74)	−2.359	0.018
Hcy (μmol/L)	15.40 (12.00, 19.70)	13.10 (11.00, 17.70)	−2.110	0.035
CK (U/L)	138.00 (73.50, 582.32)	73.00 (54.86, 95.38)	−5.665	<0.001
CK-MB (U/L)	16.65 (10.69, 41.92)	9.70 (7.63, 13.62)	−6.336	<0.001
**Echocardiography**				
LA (mm)	35.00 (33.00, 39.00)	35.00 (32.00, 37.00)	−2.106	0.035
LVEDD (mm)	49.00 (47.00, 53.00)	49.00 (46.00, 53.00)	−0.731	0.465
LVESD (mm)	34.00 (31.00, 37.00)	32.00 (29.00, 34.00)	−3.074	0.002
LVEF (%)	59.00 (54.00, 65.00)	64.00 (60.00, 69.00)	−4.810	<0.001
**Coronary angiography**				
1 vessel	71 (33.8)			
2 vessels	57 (27.1)			
3 vessels	82 (39.1)			
**Adipocytokines (pg/mL)**				
CTRP1	654.50 (584.00, 737.50)	591.50 (503.25, 669.67)	−3.321	0.001
CTRP2	342.85 (292.34, 404.74)	382.00 (337.20, 453.58)	−4.246	<0.001
CTRP3	696.50 (604.75, 826.25)	896.00 (659.75, 1132.25)	−4.494	<0.001
CTRP5	183.30 (145.73, 231.68)	176.88 (104.65, 210.28)	−2.609	0.009
CTRP9	119.75 (102.50, 135.10)	124.53 (110.26, 171.74)	−2.436	0.015
CTRP12	433.95 (333.93, 497.03)	468.45 (390.95, 565.63)	−2.980	0.003
CTRP13	493.05 (443.75, 554.75)	550.00 (491.98, 645.25)	−4.278	<0.001
CTRP15	65.95 (58.13, 74.45)	75.83 (60.76, 104.08)	−3.456	0.001
TNF-α	391.35 (330.83, 440.83)	351.55 (265.13, 437.55)	−2.570	0.010
ADP	1543.75(1273.88,1878.13)	1685.50(1482.50,2374.50)	−3.148	0.002

ACS, acute coronary syndrome; BMI, body mass index; DM, diabetes mellitus; MetS, metabolic syndrome; CAD, coronary artery disease; UA, unstable angina; NSTEMI, non-ST-segment elevation myocardial infarction; STEMI, ST-segment elevation myocardial infarction; WBC, white blood cell; TC, total cholesterol; TG, triglycerides; LDL-C, low-density lipoprotein cholesterol; HDL-C, high-density lipoprotein cholesterol; ALB, albumin; Cr, creatinine; Hcy, homocysteine; CK, creatine kinase; CK-MB, creatine kinase isoenzyme MB; LA, left atrium; LVEDD, left ventricular end-diastolic diameter; LVESD, left ventricular end-systolic diameter; LVEF, left ventricular ejection fraction; CTRP, C1q/TNF-related protein; TNF-α, tumor necrosis factor-α; ADP, adiponectin.

Patients with ACS showed significant differences in white blood cell (WBC) count, platelet count, TG, HDL-C, ALB, Cr, serum uric acid, Hcy, creatine kinase (CK), CK-MB, left atrial (LA) diameter, left ventricular end-systolic diameter (LVESD), and left ventricular ejection fraction (LVEF) (*p* < 0.05). In addition, the angiographic findings showed that coronary stenosis with three-vessels involvement (39.1%) was the most common.

**Figure 1:**
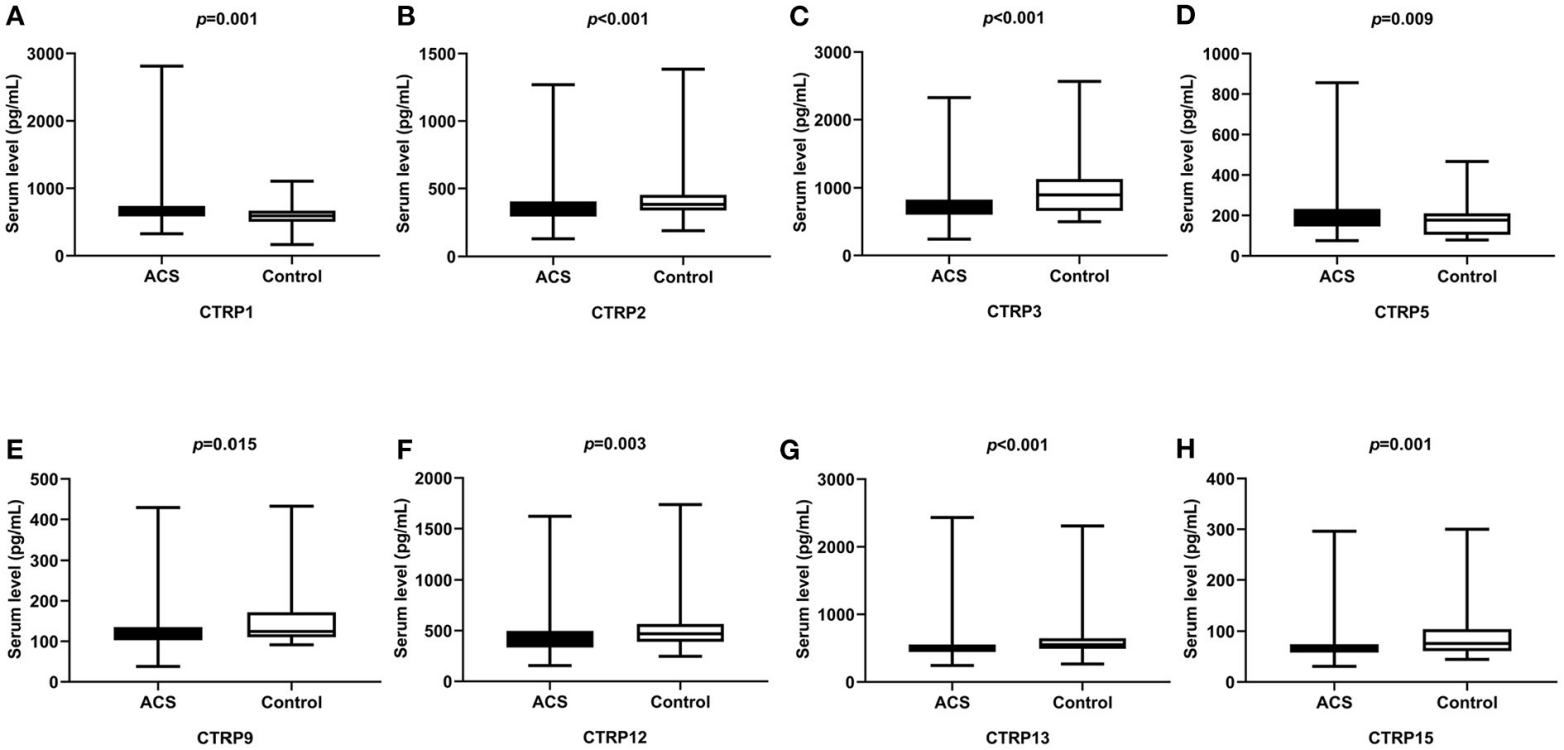
Comparison of serum CTRP family levels between ACS and control groups. **(A)** CTRP1, **(B)** CTRP2, **(C)** CTRP3, **(D)** CTRP5, **(E)** CTRP9, **(F)** CTRP12, **(G)** CTRP13, and **(H)** CTRP15.

Moreover, serum CTRP1, CTRP5, and TNF-α levels were significantly higher, whereas CTRP2, CTRP3, CTRP9, CTRP12, CTRP13, CTRP15, and ADP levels were significantly lower in the ACS group than in the Control group (*p* < 0.05, Table 1, Figure 1).

### ROC curve analyses of adipocytokines for diagnosing ACS

**Figure 2:**
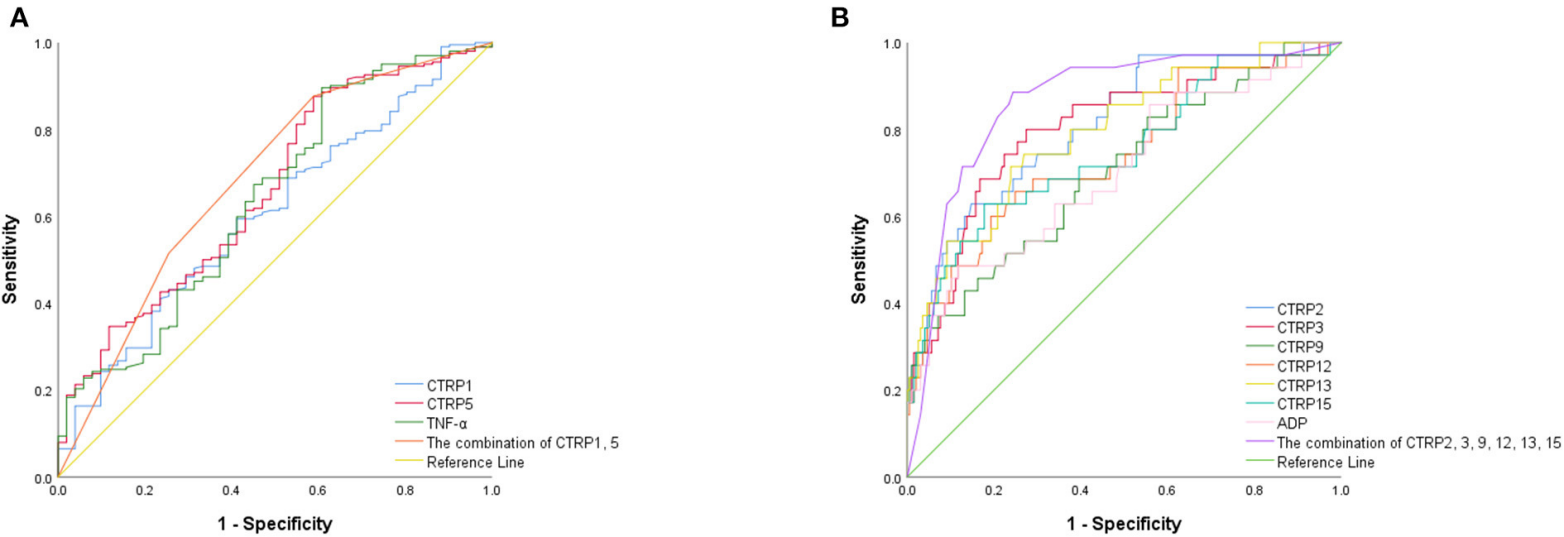
ROC curves of **(A)** pro-inflammatory and **(B)** anti-inflammatory adipocytokines for predicting ACS.

Figure 2 presents the ROC curve analyses to determine the optimal cutoff values of adipocytokines for predicting ACS. The area under the curve (AUC) of CTRP1, CTRP15, and TNF-α were 0.606 [95% confidence interval (CI): 0.521, 0.690], 0.661 (95% CI: 0.577, 0.744), 0.641 (95% CI: 0.553, 0.729). The AUC of CTRP1 + CTRP5 was 0.684 (95% CI: 0.598, 0.770). The predictive power for ACS improved after values for CTRP1 and 5 were combined (all *p* < 0.005).

The AUC of CTRP2, CTRP3, CTRP9, CTRP12, CTRP13, CTRP15, and ADP were 0.810 (95% CI: 0.732,0.888), 0.800 (95% CI: 0.715, 0.886), 0.706 (95% CI: 0.608, 0.804), 0.740 (95% CI: 0.643, 0.837), 0.794 (95% CI: 0.711, 0.877), 0.748 (95% CI: 0.651,0.845), and 0.704 (95% CI: 0.603,0.805). Furthermore, the predictive power for ACS improved after data for CTRP2, CTRP3, CTRP9, CTRP12, CTRP13, and CTRP15 were combined (AUC: 0.864; 95% CI: 0.798, 0.929; all *p* < 0.001; Table 2).

**Table 2:** ROC curve analyses of pro-inflammatory and anti-inflammatory adipocytokines for diagnosing ACS.

**Variables**	**AUC**	**95% CI**	***p*-value**	**Se (%)**	**Sp (%)**	**Cut-off**
**Pro-inflammatory adipocytokines (pg/mL)**						
CTRP1	0.606	0.521, 0.690	0.020	56.2	66.7	642.75
CTRP5	0.661	0.577, 0.744	<0.001	86.1	37.1	121.80
TNF-α	0.641	0.553, 0.729	0.002	88.5	38.2	285.60
The combination of CTRP1, 5	0.684	0.598, 0.770	<0.001	51.7	79.1	–
**Anti-inflammatory adipocytokines (pg/mL)**						
CTRP2	0.810	0.732, 0.888	<0.001	84.4	44.7	328.55
CTRP3	0.800	0.715, 0.886	<0.001	64.6	72.7	785.25
CTRP9	0.706	0.608, 0.804	<0.001	30.4	90.3	160.08
CTRP12	0.740	0.643, 0.837	<0.001	87.2	35.1	366.58
CTRP13	0.794	0.711, 0.877	<0.001	54.5	72.7	543.50
CTRP15	0.748	0.651, 0.845	<0.001	50.0	79.0	76.23
ADP	0.704	0.603, 0.805	<0.001	79.7	44.4	1,479.75
The combination of CTRP 2, 3, 9, 12, 13, and 15	0.864	0.798, 0.929	<0.001	81.4	73.9	–

ROC, receiver operating characteristic; ACS, acute coronary syndrome; CTRP, C1q/TNF-related protein; TNF-α, tumor necrosis factor-α; ADP, adiponectin.

### Univariate and multivariate logistic regression analyses of ACS risks

Table 3 presents the results of univariate and multivariate analyses using logistic regression models to determine independent predictors for ACS. After adjusting for confounding factors in Model 3, we found that CTRP family were independently associated with ACS. The OR of CTRP1, CTRP2, CTRP3, CTRP5, CTRP9, CTRP12, CTRP13, and CTRP15 expression levels per 1-SD increase were 2.692 (95% CI: 1.324, 5.471; *p* < 0.05), 0.668 (95% CI: 0.471, 0.947; *p* < 0.05), 0.350 (95% CI: 0.201, 0.609; *p* < 0.001), 1.858 (95% CI: 1.089, 3.171; *p* < 0.05), 0.661 (95% CI: 0.449, 0.973; *p* < 0.05), 0.653 (95% CI: 0.468, 0.910; *p* < 0.05), 0.716 (95% CI: 0.514, 0.999; *p* < 0.05), and 0.696 (95% CI: 0.498, 0.972; *p* < 0.05). CTRP1, as a pro-inflammatory adipokines, had a greater causative effect on ACS. Whereas, CTRP3 has a greater protective effect on ACS than other anti-inflammatory adipokines.

**Table 3:** Univariate and multivariate logistic regression analyses of ACS risks according to 1-SD increasing of serum CTRP family levels.

**CTRP family (per 1-SD increase)**	**Model 1**		**Model 2**		**Model 3**	
	**OR (95% CI)**	***p*-value**	**OR (95% CI)**	***p*-value**	**OR (95% CI)**	***p*-value**
CTRP1	2.351 (1.304, 4.236)	0.004	2.368 (1.279, 4.385)	0.006	2.692 (1.324, 5.471)	0.006
CTRP2	0.649 (0.500, 0.843)	0.001	0.692 (0.510, 0.938)	0.018	0.668 (0.471, 0.947)	0.024
CTRP3	0.502 (0.370, 0.681)	<0.001	0.487 (0.334, 0.710)	<0.001	0.350 (0.201, 0.609)	<0.001
CTRP5	1.756 (1.147, 2.688)	0.010	1.678 (1.079, 2.609)	0.022	1.858 (1.089, 3.171)	0.023
CTRP9	0.611 (0.458, 0.816)	0.001	0.632 (0.458, 0.873)	0.005	0.661 (0.449, 0.973)	0.036
CTRP12	0.730 (0.571, 0.934)	0.012	0.742 (0.562, 0.981)	0.036	0.653 (0.468, 0.910)	0.012
CTRP13	0.735 (0.577, 0.937)	0.013	0.745 (0.555, 1.000)	0.050	0.716 (0.514, 0.999)	0.049
CTRP15	0.635 (0.486, 0.829)	0.001	0.680 (0.506, 0.913)	0.010	0.696 (0.498, 0.972)	0.034

Model 1: Unadjusted.

Model 2: Adjusted for sex, age, BMI, heart rate at admission, blood pressure at admission.

Model 3: Adjusted for terms in Model 2 and smoking, dyslipidemia, hypertension, DM, MetS, history of stroke, family history of CAD.

ACS, acute coronary syndrome; CTRP, C1q/TNF-related protein; BMI, body mass index; DM, diabetes mellitus; MetS, metabolic syndrome; CAD, coronary artery disease.

**Figure 3:**
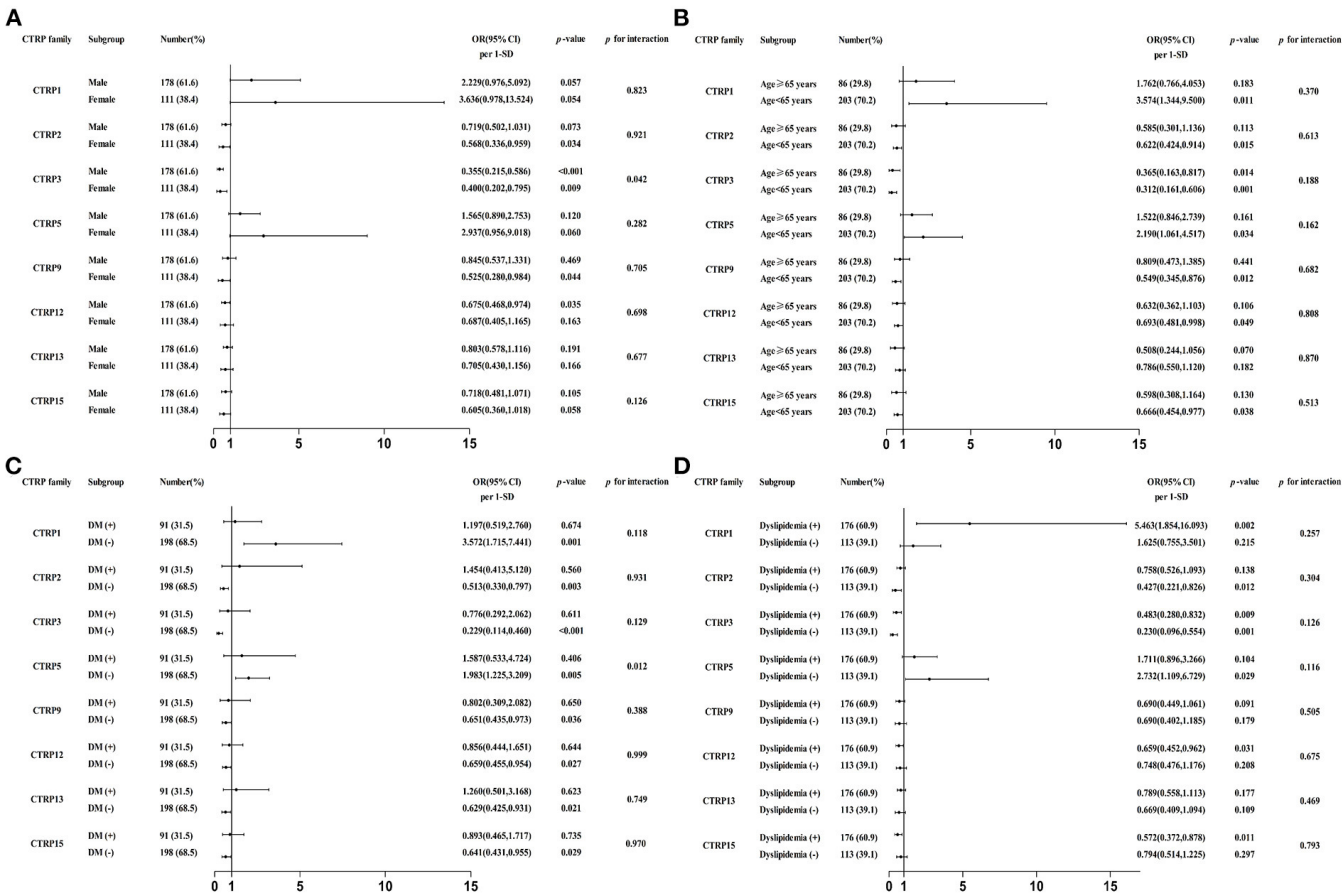
Forest graphs based on subgroup analysis for the effect of CTRP family in patients with ACS. **(A)** Subgroup analysis according to sex of patients, adjusted for age, dyslipidemia, hypertension, and diabetes mellitus (DM). **(B)** Subgroup analysis according to age of patients, adjusted for smoking, dyslipidemia, hypertension, and DM. **(C)** Subgroup analysis according to DM status of patients, adjusted for sex, smoking, dyslipidemia, and hypertension. **(D)** Subgroup analysis according to dyslipidemia status of patients, adjusted for sex, smoking, hypertension, and DM.

Subsequently, the independent association between CTRP family and ACS was also assessed in various subgroups according to sex (male or female), age (≥ 65 or <65 years), and DM and dyslipidemia status (with or without). The CTRP3 expression level exhibited a higher protective role against ACS in male than in female patients [OR (95% CI) per 1-SD: 0.355 (0.215, 0.586), *p* < 0.001 vs. 0.400 (0.202, 0.795), *p* < 0.05, respectively; *p* for interaction < 0.05]. Moreover, the CTRP5 expression level was an independent risk factor for ACS in patients without, however, not in those with DM [OR (95% CI) per 1-SD: 1.983 (1.225, 3.209), *p* < 0.05 vs. 1.587 (0.533, 4.724), *p* = 0.406, respectively; *p* for interaction < 0.05]. The specific subgroup analysis results are illustrated in Figure 3.

Supplementary Table 1 shows the association between the tertiles of serum CTRP family expression levels and ACS. The risk of ACS increased gradually with rising CTRP1 tertiles [OR (95% CI): T1: reference, T2: 1.828(0.849, 3.939), T3: 3.375 (1.432, 7.956); *p* for trend < 0.05]. In contrast, the risk of ACS showed a downward trend with increasing CTRP2, CTRP12, and CTRP13 expression levels (*p* for trend < 0.05). However, the protective role of CTRP3 and CTRP15 expression for ACS exhibited a U-shaped trend with increasing levels (*p* for trend < 0.05).

### Correlation analysis of CTRP family and the severity of coronary artery stenosis

The results illustrated in Table 4 demonstrate that the serum levels of CTRP1 were positively correlated, whereas those of CTRP2, CTRP13, and CTRP15 were negatively correlated with the Gensini score (*p* < 0.05).

**Table 4:** Correlation analysis of CTRP family and Gensini score.

**CTRP family (pg/mL)**	**Gensini score**	
	***r***	***p*-value**
CTRP1	0.224	<0.001
CTRP2	−0.179	0.002
CTRP3	−0.114	0.063
CTRP5	0.092	0.125
CTRP9	−0.102	0.106
CTRP12	−0.113	0.057
CTRP13	−0.153	0.009
CTRP15	−0.217	<0.001

CTRP, C1q/TNF-related protein.

**Figure 4:**
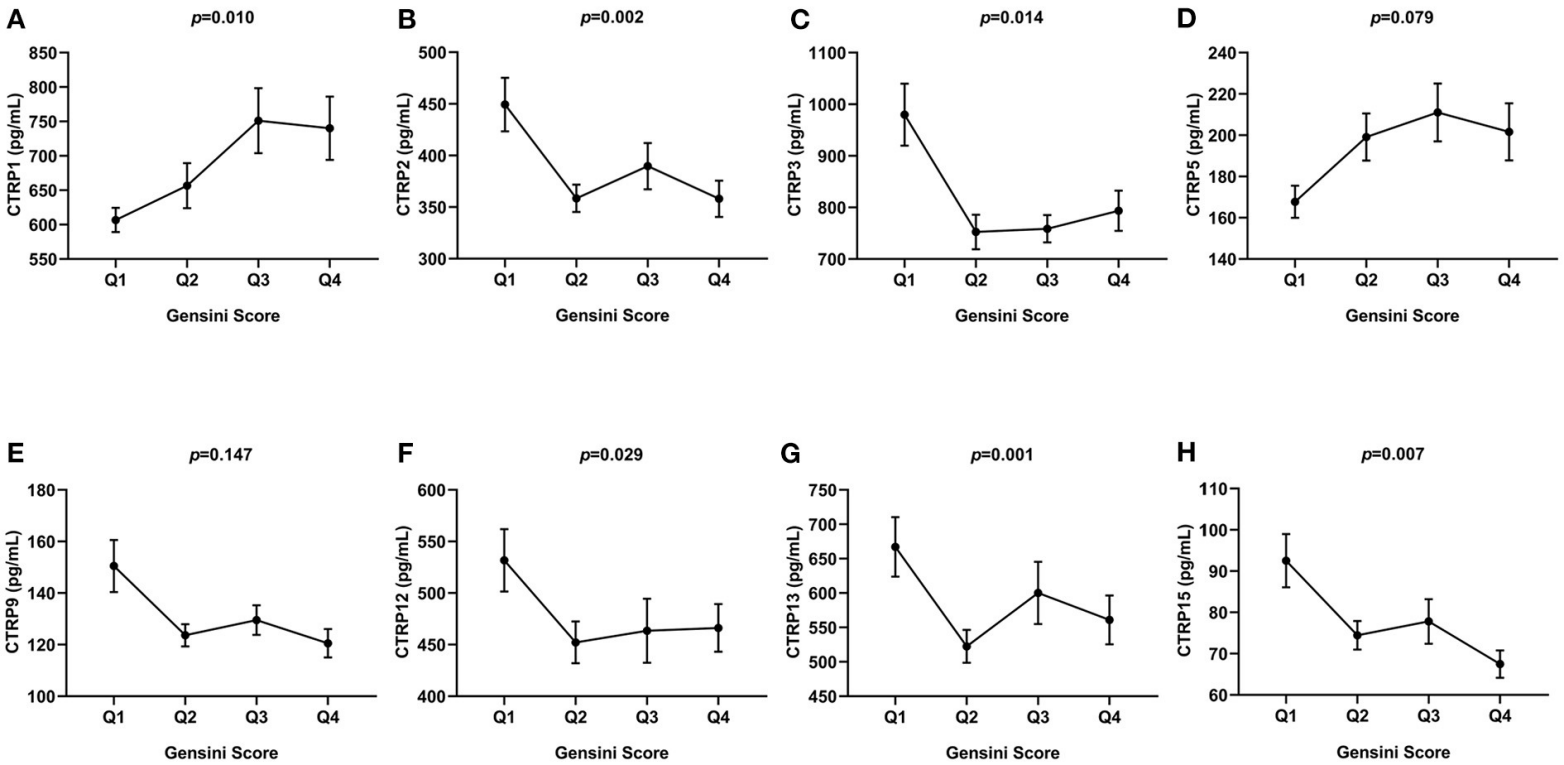
Comparison of serum CTRP family levels between different groups according to Gensini score quartiles. **(A)** CTRP1, **(B)** CTRP2, **(C)** CTRP3, **(D)** CTRP5, **(E)** CTRP9, **(F)** CTRP12, **(G)** CTRP13, and **(H)** CTRP15. Data are mean ± standard error of the mean (SEM).

In Figure 4, patients were divided into four quartiles according to the Gensini score, Q1 (*n* = 81, Gensini score ≤ 26.5), Q2 (*n* = 64, 26.5 < Gensini score ≤ 39), Q3 (*n* = 73, 39 < Gensini score ≤ 62.5), and Q4 (*n* = 71, Gensini score > 62.5). The expression level of CTRP1 was increased across the quartiles of the Gensini score. In addition, CTRP2, CTRP3, CTRP12, CTRP13, and CTRP15 levels showed a decreasing trend across the quartiles of Gensini score (*p* < 0.05, Figure 4).

## Discussion

In the present study, we investigated the association between CTRP family and patients with ACS. The major findings of our study were (1) CTRP family members, as pro-inflammatory or anti-inflammatory adipokines, were independent predictors for ACS; (2) the association between CTRP family and ACS was not significantly affected by sex, age, DM, and dyslipidemia status; (3) increasing levels of CTRP1, CTRP2, CTRP12, and CTRP13 showed a linear trend with the risk of ACS; and (4) the serum levels of CTRP1, CTRP2, CTRP13, and CTRP15 showed a weak correlation with the severity of coronary artery stenosis. To the best of our knowledge, compared with previous studies, this is the first study to elucidate and compare the diagnostic performance and causative risk of eight CTRP family members for ACS in the general population and different subgroups.

As the leading cause of ACS, atherosclerosis is a complex process, and its development involves lipids, immune cells, vascular smooth muscle cells (VSMCs), and various adipokines (11). Previous studies have reported the diagnostic and prognostic roles of novel adipokines in patients with ACS (12). CTRP family members, mostly secreting from the EAT, can affect atherosclerotic plaques through different mechanisms.

A previous study showed that serum CTRP1 levels were higher in patients with CAD than in those without CAD, which can promote the expression of adhesion molecules and synthesis of inflammatory cytokines through activating the p38 mitogen-activated protein kinase (MAPK)/nuclear factor (NF)-kB pathway (13). Our study showed CTRP1 as an independent risk factor for ACS, and increasing tertiles of serum levels, significantly increased ACS risks, further indicating the pro-inflammatory and pro-atherogenic effects of CTRP1. In the present study, we demonstrated that patients with ACS had higher serum CTRP5 levels than those without ACS, and elevated serum levels were associated with the increased risk of ACS. Mechanistically, CTRP5 can exert pro-inflammatory effects by promoting LDL trafficking and oxidation through upregulation of 12/15-lipoxygenase expression (14). Moreover, CTRP5 facilitates the growth, migration, and inflammation of VSMCs by multiple pathways causing in-stent restenosis after coronary stent implantation (15). The above studies illustrate the close relationship between CTRP5 and ACS. Furthermore, the association between CTRP5 and a wide range of diseases has been reported, such as heart failure (16), DM (17), MetS (18), and retinal degeneration (19). In our study, we found that the causative effect of CTRP1 for ACS was significantly greater than that of CTRP5.

CTRP2 has more similar biological activities to those of ADP than other CTRP family members do (6). Previous studies have reported that mice overexpressing CTRP2 exhibited improved insulin resistance and were better able to deal with acute lipid challenges than the control mice (20). In contrast, Lei et al. (21) found that CTRP2 knockout mice had significantly elevated plasma TG and VLDL-TG, and the absence of CTRP2 promoted hepatic TG secretion. Thus, we speculated that CTRP2 exerts anti-inflammatory effects through modulating glucose and lipid metabolism. In our study, we found that the concentration of CTRP2 was significantly lower in the ACS group, and the risk of ACS decreased with increasing CTRP2 levels. More importantly, the protective effect of CTRP2 on ACS remained consistent in the different subgroups (*p* for interaction > 0.05). However, Ilbeigi et al. (22) found that CTRP2 levels were higher in patients with CAD than in those without CAD. Therefore, more clinical studies with large sample size are necessary to obtain more accurate results.

The contribution of CTRP3 and CTRP9 to the pathogenesis of ACS is supported by *in vitro* and *vivo* observations. CTRP3 alleviated oxidized (ox)-LDL-induced inflammation and endothelial dysfunction by activating the phosphoinositide 3-kinase (PI3K)/Akt/endothelial nitric oxide synthase (eNOS) pathway (23). Moreover, ox-LDL can be identified by macrophages and causes foam cell formation, which plays a pivotal role in the progression of atherosclerosis (24). Similarly, CTRP9 exerts its anti-inflammatory action through inhibiting vascular inflammation, regulating lipid metabolism, and limiting VSMCs migration (25). Serum levels of CTRP9 were reported to be lower in patients with CAD than in those without CAD, and the expression of CTRP9 mRNA decreased significantly in the EAT of CAD patients (26). In summary, CTRP3 and CTRP9 play a protective role against atherosclerosis development, which is consistent with our findings that elevated levels of both were independent protective factors for ACS. Furthermore, the protective effect of CTRP3 on ACS was significantly greater than that of other anti-inflammatory CTRP family members.

CTRP12, which is known as adipolin, is mainly expressed in adipose tissue. In this study, we found that elevated serum CTRP12 levels were an independent protective factor for ACS. Several previous studies have reported CTRP12 levels to be significantly lower in patients with CAD than in those without CAD and exhibit an independent association with the risk of CAD (27, 28). These findings support our present observations. Regarding the potential mechanisms, Wang et al. (29) found that CTRP12 inhibits atherosclerosis by promoting cholesterol efflux from macrophages and alleviating inflammatory response. In a previous study, CTRP13 promoted autophagy in macrophages and inhibit lipid ingestion by macrophage ingestion of lipid, thereby exerting protective effects on atherosclerosis. Wang et al. (30) showed that CTRP13 levels in the CAD group were lower than those in the control group, which is consistent with our study findings. Additionally, we demonstrated a significantly negative linear association between CTRP12 and CTRP13 levels and the risk of ACS, which further confirmed their protective effects in patients with ACS.

As a recently discovered member of the CTRP family, CTRP15 (myonectin) is mainly expressed in the muscle tissues. In our study, we found CTRP15 to be an independent protective factor for ACS, which is consistent with the findings of Zhang et al. (31). Mechanistically, CTRP15 can regulate inflammation, mainly *via* reducing interleukin (IL)-6 and TNF-α secretions from macrophages (32). Furthermore, *in vivo* experiments have shown that CTRP15 increased serum HDL-C levels and promoted macrophage cholesterol efflux, thereby ameliorating lipid accumulation and atherosclerosis (33). Shokoohi et al. (34) reported that the serum levels of CTRP15 were elevated in patients with CAD. Interestingly, in our study, we found that the protective effects of CTRP15 against ACS exhibited a trend of decreasing firstly and then increasing (U-shaped trend). Future research is needed to elucidate the association between CTRP15 and patients with ACS.

Moreover, we present an in-depth analysis of the effect of CTRP family members in different subgroups of patients with ACS, which has not been well-explored in previous studies. Compared to the general population, the causative or protective effect of the CTRP family members on ACS remained consistent, because no significant difference was identified across most subgroups (*p* for interaction > 0.05). However, the protective effect of CTRP3 appeared stronger in male patients than in female patients. Wagner et al. (35) also found a difference in CTRP3 expression levels in male and female patients. The mechanism behind this sex-related difference is speculated to involve hormonal status. In addition, subgroup analysis showed differences in the effect of CTRP5 in patients with or without DM. Liu et al. (17) reported that CTRP5 can cause dysfunction of the vascular endothelium in patients with DM, who also had higher levels of the protein. Therefore, we speculated that the increase of CTRP5 levels in patients with ACS and DM would be affected by glucose metabolism, thereby weakening the diagnostic usefulness of CTRP5 for ACS.

In our study, we found that the serum levels of CTRP1, CTRP2, CTRP13, and CTRP15 showed a weak correlation with the Gensini score. CTRP family members showed an apparent increasing or decreasing trend with the Gensini score quartiles. In contrast to previous studies, which used the number of lesion vessels, we used the Gensini score to systemically evaluate the association between CTRP family members and the severity of coronary artery stenosis. This suggests the potential usefulness of CTRP family members, especially CTRP1 and CTRP15, in assessing the severity of coronary artery stenosis.

Our study has some limitations. First, our data from patients were from a single-center, and there may have selection bias and limit the generalizability of our findings to patients with ACS. In addition, the cutoff value of the expression level of CTRP family members may vary according to the patient population. Thus, the cutoff value may not be applicable in patients with other cardiovascular diseases or in patients with ACS from other countries. Second, the serum levels of CTRP family members were evaluated only once after patients were admitted, and information on the change in levels during hospitalization was limited. Third, this is a cross-sectional study. Therefore, this study could not determine the causality between CTRP family and ACS. More large-scale prospective studies should be conducted to validate the diagnostic significance of CTRP family members in patients with ACS.

## Conclusion

In this study, we discovered that CTRP family members as risk or protective factors, were independently associated with ACS. Specifically, CTRP1 and CTRP5 were identified as independent risk factors for ACS, whereas CTRP2, CTRP3, CTRP9, CTRP12, CTRP13, and CTRP15 were independent protective factors for ACS. The causative effect of CTRP1 on ACS and the protective effect of CTRP3 on ACS were significantly higher than that of other CTRP family members. Moreover, CTRP1 and CTRP15 were more closely correlated with the severity of coronary artery stenosis than other CTRP family members were. Finally, CTRP family members have the potential to serve as novel diagnostic biomarkers and future therapeutic targets of ACS in clinical practice.

## Data availability statement

The raw data supporting the conclusions of this article will be made available by the authors, without undue reservation.

## Ethics statement

The studies involving human participants were reviewed and approved by Ethics Committee of Chengde Medical University Affiliated Hospital. The patients/participants provided their written informed consent to participate in this study.

## Author contributions

YL, CW, and LS designed the study. EX designed the experimental methodology. ZD contributed to data collection. ZZ, FS, YT, YZ, and WF analyzed and interpreted the data. YL and LS wrote, reviewed, and edited the manuscript. All authors contributed to manuscript revision, read, and approved the submitted version.

## Funding

This research was supported by grants from the Natural Science Foundation of Hebei Province (Grant no. H2021406071) awarded to LS and the Department of Education Graduate Innovation Funding of Hebei Province (Grant no. CXZZSS2022139) awarded to YL.

## Conflict of interest

The authors declare that the research was conducted in the absence of any commercial or financial relationships that could be construed as a potential conflict of interest.

## Publisher's note

All claims expressed in this article are solely those of the authors and do not necessarily represent those of their affiliated organizations, or those of the publisher, the editors and the reviewers. Any product that may be evaluated in this article, or claim that may be made by its manufacturer, is not guaranteed or endorsed by the publisher.
