# Supplementary material for: Role of serum C1q/TNF-related protein family levels in patients with acute coronary syndrome
Source: Front Cardiovasc Med. 2022 Aug 19;9:967918. doi: 10.3389/fcvm.2022.967918 (PMC9437344; doi:10.3389/fcvm.2022.967918)
Supplement: Supplementary file 1 [file Table_1.docx]

Supplementary Material

**Supplementary Table 1** Univariate and multivariate logistic regression analyses of ACS risks according to tertiles of serum CTRP family levels

| CTRP family  (tertiles 1–3) | | Model 1 | | Model 2 | | Model 3 | |
| --- | --- | --- | --- | --- | --- | --- | --- |
|  |  | OR (95%CI) | *p*-value | OR (95%CI) | *p*-value | OR (95%CI) | *p*-value |
| CTRP1 | T1 | 1 (Reference) | - | 1 (Reference) | - | 1 (Reference) | - |
|  | T2 | 1.729 (0.909, 3.286) | 0.095 | 1.701 (0.854, 3.388) | 0.131 | 1.828 (0.849, 3.939) | 0.123 |
|  | T3 | 3.036 (1.490, 6.186) | 0.002 | 3.073 (1.411, 6.693) | 0.005 | 3.375 (1.432, 7.956) | 0.005 |
|  | *p* for trend | 0.002 |  | 0.004 |  | 0.005 |  |
| CTRP2 | T1 | 1 (Reference) | - | 1 (Reference) | - | 1 (Reference) | - |
|  | T2 | 0.329 (0.156, 0.694) | 0.004 | 0.414 (0.188, 0.910) | 0.028 | 0.412 (0.174, 0.975) | 0.044 |
|  | T3 | 0.237 (0.114, 0.493) | <0.001 | 0.281 (0.128, 0.617) | 0.002 | 0.231 (0.095, 0.561) | 0.001 |
|  | *p* for trend | <0.001 |  | 0.002 |  | 0.001 |  |
| CTRP3 | T1 | 1 (Reference) | - | 1 (Reference) | - | 1 (Reference) | - |
|  | T2 | 1.436(0.620,3.326) | 0.398 | 1.612(0.661,3.930) | 0.293 | 1.306(0.493,3.457) | 0.591 |
|  | T3 | 0.262(0.131,0.523) | <0.001 | 0.317(0.149,0.673) | 0.003 | 0.155(0.060,0.401) | <0.001 |
|  | *p* for trend | <0.001 |  | 0.001 |  | <0.001 |  |
| CTRP5 | T1 | 1 (Reference) | - | 1 (Reference) | - | 1 (Reference) | - |
|  | T2 | 1.322 (0.698, 2.502) | 0.392 | 1.066 (0.528, 2.151) | 0.859 | 1.448 (0.660, 3.176) | 0.355 |
|  | T3 | 1.999 (1.007, 3.967) | 0.048 | 1.726 (0.827, 3.603) | 0.146 | 2.010 (0.884, 4.568) | 0.096 |
|  | *p* for trend | 0.048 |  | 0.149 |  | 0.095 |  |
| CTRP9 | T1 | 1 (Reference) | - | 1 (Reference) | - | 1 (Reference) | - |
|  | T2 | 0.694 (0.298, 1.613) | 0.396 | 0.619 (0.239, 1.603) | 0.324 | 0.515 (0.173, 1.532) | 0.233 |
|  | T3 | 0.468 (0.209, 1.051) | 0.066 | 0.441 (0.180, 1.079) | 0.073 | 0.564 (0.207, 1.540) | 0.264 |
|  | *p* for trend | 0.063 |  | 0.072 |  | 0.299 |  |
| CTRP12 | T1 | 1 (Reference) | - | 1 (Reference) | - | 1 (Reference) | - |
|  | T2 | 0.740 (0.374, 1.464) | 0.388 | 0.654 (0.315, 1.356) | 0.254 | 0.416 (0.179, 0.969) | 0.042 |
|  | T3 | 0.416 (0.216, 0.799) | 0.009 | 0.348 (0.171, 0.708) | 0.004 | 0.174 (0.075, 0.403) | <0.001 |
|  | *p* for trend | 0.008 |  | 0.003 |  | <0.001 |  |
| CTRP13 | T1 | 1 (Reference) | - | 1 (Reference) | - | 1 (Reference) | - |
|  | T2 | 0.439 (0.209, 0.921) | 0.029 | 0.498 (0.225, 1.105) | 0.087 | 0.308 (0.126, 0.754) | 0.010 |
|  | T3 | 0.225 (0.110, 0.459) | <0.001 | 0.184 (0.084, 0.403) | <0.001 | 0.120 (0.049, 0.293) | <0.001 |
|  | *p* for trend | <0.001 |  | <0.001 |  | <0.001 |  |
| CTRP15 | T1 | 1 (Reference) | - | 1 (Reference) | - | 1 (Reference) | - |
|  | T2 | 1.148 (0.554, 2.378) | 0.711 | 1.317 (0.593, 2.926) | 0.498 | 1.559 (0.646, 3.766) | 0.323 |
|  | T3 | 0.372 (0.194, 0.713) | 0.003 | 0.370 (0.178, 0.772) | 0.008 | 0.360 (0.159, 0.818) | 0.015 |
|  | *p* for trend | 0.002 |  | 0.004 |  | 0.007 |  |

Model 1: Unadjusted.

Model 2: Adjusted for sex, age, BMI, heart rate at admission, blood pressure at admission.

Model 3: Adjusted for terms in Model 2 and smoking, dyslipidemia, hypertension, DM, MetS, history of stroke, family history of CAD.

ACS, acute coronary syndrome; CTRP, C1q/TNF-related protein; BMI, body mass index; DM, diabetes mellitus; MetS, metabolic syndrome; CAD, coronary artery disease.
